# Supplementary figures and images for: Diverse stimuli engage different neutrophil extracellular trap pathways
Source: eLife. 2017 Jun 2;6:e24437. doi: 10.7554/eLife.24437 (PMC5496738; doi:10.7554/eLife.24437)

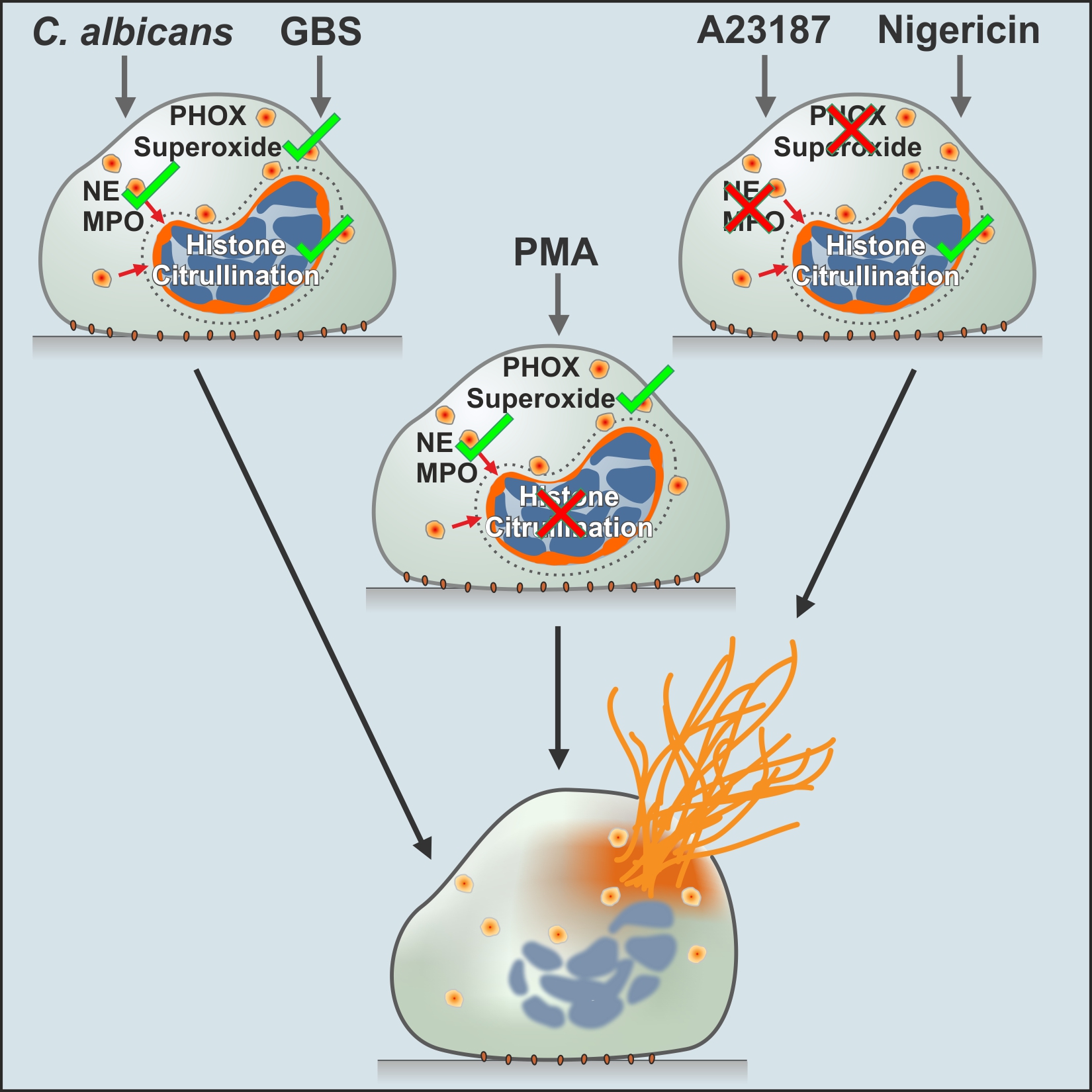

Supplement: Supplementary file 1. — In this study, we investigated whether NETosis occurs through a single signalling pathway or is induced by the five stimuli of interest in a diverse manner. As demonstrated, NETosis in response to C. albicans and GBS requires ROS, MPO and NE and induces histone H3 citrullination. This is in comparison to the NETosis seen in response to A23187 and nigericin during which none of the molecules highlighted above are required but citrullination of histone H3 does occur. Finally, we re-confirm that PMA-induced NETosis requires ROS, MPO and NE but does not result in the citrullination of histone H3. DOI: http://dx.doi.org/10.7554/eLife.24437.030 [file elife-24437-supp1.jpg]
